# Being an Imposter: Growing Out of Impostership

**DOI:** 10.31729/jnma.5505

**Published:** 2020-12-31

**Authors:** Sumina Mainali

**Affiliations:** 1 Kathmandu University School of Medical Sciences, Dhulikhel, Kavre, Nepal

**Keywords:** *failure*, *fraudulence*, *impostorism*, *medical student*, *self-doubt*

## Abstract

Imposter syndrome is the fear of being discovered as an intellectual fraud. It is an internal experience of believing that you are not as competent as others perceive you to be. It lowers person's inner self-confidence, self-esteem that impedes work performance and this phenomenon is more prevalent among medical students. However, talking openly about it with peers, rooting positive thoughts and building confidence helps us to appropriate reality of our situation and counter our negative self-talk.

## INTRODUCTION

Imposter syndrome (also known as Imposter phenomena, perceived fraudulence or imposter experience) describes high achieving individuals who despite their objective successes, fail to internalize their accomplishments and have persistent feelings of self-doubt and fear of being exposed as a fraud or imposter.^1^

It is a phenomenon, an experience; not a recognized psychiatric disorder. Imposter syndrome is not featured in American Psychiatric Association's Diagnostic and statistical Manual nor is listed as a diagnosis in the International Classification of Diseases.^2^

Psychologists Dr. Pauline Rose Clance and Suzanne Imes first described this syndrome in 1978 in the paper "The imposter phenomenon in high achieving women Dynamics and therapeutic intervention"^3^ which theorized that professional women were uniquely affected by imposter syndrome. Later, Clance concluded that anyone who is unable to internalize and acknowledge one's success may have imposter syndrome, in her book “when success makes you feel fake”

## IDENTIFICATION OF IMPOSTERS

Negative thinking, self-doubt, and self-sabotaging one's own successes are characteristic behaviors of those suffering from imposter syndrome. They often attribute any success they have achieved to luck or perfect timing. Feelings of low self-esteem and lack of confidence are also common. They live in fear that they won't live up to expectations and will ultimately be exposed as a fraud. Imposter set very challenging goals and feel disappointed when they fall short.

Despite high achievements on standardized tests, praise and acclaim from colleagues and teachers, I did not experience an internal sense of success. I secretly doubted my intellect and explained failure with lack of ability instead of attributing failure to task difficult. All these experiences made me comprehend that I had an imposter syndrome.

## MY OWN STRUGGLE

In today's world, where we are chasing success, we frequently encounter the situation that perpetuate our self-doubting nature and nourish the feeling of being an imposter from within. An estimated seventy percent of people experience feeling like an imposter at some point in their lives.^4^

During the medical school, as we navigate the vast knowledge we are expected to absorb and begin to develop clinical skills, we may experience an increase of imposter syndrome-As intrinsic and extrinsic expectations regarding our performance increase, the fear of being exposed as a fraud also increases.^5^

Imposter syndrome exists in a significant percent of medical students and appears to peak in 3rd year of medical school.^6^ Third-year medical students identified most strongly with items related to unfounded fear of failure, hesitance to share recognition before it is announced, remembering failures rather than successes, believing themselves less capable than others, and worrying about succeeding.^7^

I can honestly say that I have struggled with this on and off throughout my life. It all started from the very beginning of my medical career when I joined pre-medical entrance preparation session. Entrance preparation while never easy had become particularly stressful in those days of my life when the negative feeling and self-doubting started invading me. Thoughts of “I am not as intelligent and creative enough compared to the people around me. I am not worthy; I don't deserve this” constantly preoccupied my mind. This ill-founded self-doubt always demotivated me and hampered my productivity. Dealing with this internal struggle and after a year of hard work and preparation I finally was enrolled into a medical school. Something that previously seemed impossible to me has now become a part of my life. However, I never got rid of this unwanted sense of insecurity. I was surrounded by seventy-five brilliant classmates with their exceptional academic qualification. I felt inferior to and left out. I felt like an outsider who did not deserve a place in this part of the world. There was always something with which they were already familiar with, but for me, it was unheard-of. To me I was the only person who did not deserve to be there, the rest were all clearly, unquestionably deserving. I believed all my achievements were down to luck or perfect timing. I always questioned my abilities and used negative self-talk to convince myself that I did not own this success.

The clinical symptoms most frequently reported are generalized anxiety, lack of self-confidence, depression and frustration related to inability to meet self-imposed standards of achievements.^3^ Imposter syndrome lowers person's self-confidence and self-esteem. It impairs work performance, career advancement and cause physical and mental problems through guilt, worry and anxiety. The competitive environment of medical school make the student realize “they are not good enough” on top of these stressors, which can lead to social isolation, academic difficulties and misaligned career path.

## OVERCOMING THE IMPOSTER SYNDROME

I admit that I am an imposter and it is not a big deal, it happens to most of us, and it depends on how we cope with this condition. In the absence of specific treatment recommendations for impostor syndrome, patients with impostor feelings should be rigorously screened for depression and anxiety and treated for these with evidence-based therapies.^1^

Recognizing the feelings of being an imposter and naming oneself as imposter makes us lean into that feeling and get to its root. Sharing our emotions with trusted colleagues help us to appreciate the reality of our situation and counter the negative self-talk. Knowing someone else feels the same as we do can offer a lot of relief and help us to stop finding fault within ourselves. We should stop comparing ourselves to others as we can never see the inside of others. Those who we perceived to be experts might just be trying to sound like experts about the things that were new to them. Evaluation of one's success, such as listing all the gained accomplishments builds up confidence. Accept who you are and embrace your strength and improve weakness. Learn to praise accomplishments and celebrate success. Even if failure accompanies one should be able to reframe those failures as a learning opportunity. Every failure should be accepted as a part of life that will help us to perform even better next time. Do not let your ill feeling stop you from pursuing your goals. Yet, if you cannot get rid of such feelings, even after you tried out everything, then it is best to speak to a mental health professional. Author Valerie Young, an expert on imposter syndrome says, “people can still have an imposter moment, but not an imposter life.”

## WAYS FORWARD

Imposter phenomenon is a ubiquitous phenomenon that anyone can feel in some facet of their lives. People who we considered, the most successful also have encountered this phenomenon according to them. Famously, Albert Einstein described himself as “involuntary swindler”. There is no such thing as an overnight success and a failure in life is inevitable. Spend less time worrying about failures and more time appreciating yourself and your deeds. The best way to deal with it is not to avoid it but to accept it as a challenge, beat it with grit and confidence. Rooting positive thoughts, talking freely about such ill feelings and breaking the silence can mitigate imposter syndrome. Every success requires consistent hardwork. We can never win over our inner critic unless we belief we are equally capable and competent to overcome every barrier that life puts forward. So never let your low self-esteem, or self-doubt derail your career.
